# HMGB1 Protein Interactions in Prostate and Ovary Cancer Models Reveal Links to RNA Processing and Ribosome Biogenesis through NuRD, THOC and Septin Complexes

**DOI:** 10.3390/cancers13184686

**Published:** 2021-09-18

**Authors:** Aida Barreiro-Alonso, Mónica Lamas-Maceiras, Lidia Lorenzo-Catoira, Mercedes Pardo, Lu Yu, Jyoti S. Choudhary, M. Esperanza Cerdán

**Affiliations:** 1 EXPRELA Group, Centro de Investigaciones Científicas Avanzadas (CICA), University of A Coruña (UDC), 15008 A Coruña, Spain; monica.lamas@udc.es (M.L.-M.); lidia.lorenzo.catoira@udc.es (L.L.-C.); 2 Instituto de Investigación Biomédica de A Coruña (INIBIC), 15006 A Coruña, Spain; 3 Department of Biology, Faculty of Sciences, Campus de A Zapateira, University of A Coruña (UDC), 15008 A Coruña, Spain; 4 Functional Proteomics, The Institute of Cancer Research, London SW7 3RP, UK; mercedes.pardocalvo@icr.ac.uk (M.P.); lu.yu@icr.ac.uk (L.Y.); jyoti.choudhary@icr.ac.uk (J.S.C.)

**Keywords:** HMGB1 interactome, ribosome biogenesis, RNA processing, ovary cancer, prostate cancer

## Abstract

**Simple Summary:**

HMGB1 over-expression is associated to prostate and ovary cancers: in this work, using a proteomic approach, we aimed to discover new protein interactions that might contribute to understand the oncogenic function of HMGB1 in cancers models. Our findings show that HMGB1 interacts with components of the NuRD, THOC and septin complexes, revealing new connections of HMGB1 functions to RNA processing and ribosome biogenesis. Results might contribute to consider the components of these interactomes as targets for diagnosis and therapy in future studies.

**Abstract:**

This study reports the HMGB1 interactomes in prostate and ovary cancer cells lines. Affinity purification coupled to mass spectrometry confirmed that the HMGB1 nuclear interactome is involved in HMGB1 known functions such as maintenance of chromatin stability and regulation of transcription, and also in not as yet reported processes such as mRNA and rRNA processing. We have identified an interaction between HMGB1 and the NuRD complex and validated this by yeast-two-hybrid, confirming that the RBBP7 subunit directly interacts with HMGB1. In addition, we describe for the first time an interaction between two HMGB1 interacting complexes, the septin and THOC complexes, as well as an interaction of these two complexes with Rab11. Analysis of Pan-Cancer Atlas public data indicated that several genes encoding HMGB1-interacting proteins identified in this study are dysregulated in tumours from patients diagnosed with ovary and prostate carcinomas. In PC-3 cells, silencing of *HMGB1* leads to downregulation of the expression of key regulators of ribosome biogenesis and RNA processing, namely *BOP1*, *RSS1*, *UBF1*, *KRR1* and *LYAR*. Upregulation of these genes in prostate adenocarcinomas is correlated with worse prognosis, reinforcing their functional significance in cancer progression.

## 1. Introduction 

The HMGB protein family includes both transcription factors, which regulate gene expression by recognising specific regulatory DNA sequences in promoters, and also proteins not classified as canonical transcription factors. The latter, which are considered architectural chromatin components that bind to DNA without sequence specificity, influence transcriptional regulation and DNA repair by different mechanisms, including nucleosome remodelling, chromatin dynamics and epigenetic modification (reviewed in [1]). Human HMGB1 binds DNA without sequence specific recognition and the gene encoding it, *HMGB1*, is overexpressed in many types of cancer [2], including those of prostate [3] and ovary [4]. Although *HMGB1* over-expression is extensively associated to cancer [5] and it has been demonstrated that *HMGB1* silencing in colorectal cancer cells slows cell growth, extends the cell proliferation cycle, and significantly inhibits the growth of xenograft tumour in nude mice [6], little is known about the mechanism underlying HMGB1’s role in oncogenesis.

Proteomic techniques allow the characterisation of protein–protein interactions and open new insights into mechanisms of biological processes and human diseases. The role of interactomics in expanding the advancement of biomedical science in cancer research and therapy is well established [7]. We have previously reported interactome studies of HMGB1 and HMGB2 proteins in prostate [8] and ovary [9] cancer cells based on the yeast two-hybrid approach, but the number of proteins identified was low. To extend the in vivo discovery in cancer cells, we have undertaken a different HMGB1-interactome analysis approach based on immunoprecipitation (IP) and mass spectrometry (MS) in PC-3 and SKOV-3 cell lines that represent models for prostate and ovarian cancer, respectively. HMGB1 has complex and diverse functions depending on its cellular location and acts as a DNA chaperone in the nucleus, but also controls apoptosis and autophagy in the cytoplasm, and acts extracellularly as an alarmin [2,10]. Combining IP-MS analysis with subcellular fractionation prior to immunoprecipitation allowed us to pinpoint the cellular localisation of the interactions identified. We found that HMGB1 interacts with components of the NuRD complex, THOC complex and septins in the nucleus. We also identified novel interactions between septins and the THOC complex, as well as their interaction with Rab11. The HMGB1 interactome is enriched in proteins involved in RNA processing and ribosome biogenesis. Interestingly, *HMGB1* silencing in PC-3 cells results in dysregulation of genes involved in these processes too, mimicking the dysregulation observed in tumours from patients diagnosed with ovary cystadenocarcinoma and prostate adenocarcinoma (acinar type) with lower levels of HMGB1.

## 2. Materials and Methods

### 2.1. Cell Culture

PC-3, DU-145 (human prostate cancer) and SKOV-3 (human ovarian cancer) cell lines were grown in RPMI-1640 (Thermo Fisher Scientific, Inc., Waltham, MA, USA) and McCoy’s 5a Modified Medium (Thermo Fisher Scientific, Inc., Waltham, MA, USA) supplemented with 10% heat-inactivated foetal bovine serum (Thermo Fisher Scientific, Inc., Waltham, MA, USA) and 1% penicillin-streptomycin (Thermo Fisher Scientific, Inc., Waltham, MA, USA), respectively. Cells were cultured at 37 °C, 5% CO_2_ in a humidified incubator. Cells were tested regularly for mycoplasma contamination.

### 2.2. Subcellular Fractionation 

Confluent SKOV-3 and PC-3 cells were pelleted down by centrifugation at 1000 rpm (Centrifuge 5804R, A-4-44 rotor, Eppendorf, Hamburg, Germany) for 5 min and cell fractionation was performed immediately. Each pellet was resuspended in Lysis Buffer (10 mM Tris-HCl pH 8.0, 10 mM NaCl, 1.5 mM MgCl_2_, 0.05% NP-40) supplemented with HaltTM Protease and Phosphatase Inhibitor Single-Use Cocktail (Thermo Fisher Scientific, Inc., Waltham, MA, USA) and 1mM DTT just prior to use and incubated for 10 min on ice. The lysate was then added carefully onto an equal volume of sucrose cushion (10 mM Tris-HCl pH 8.0, 10 mM NaCl, 1.5 mM MgCl_2_, 1 mM DTT, 0.05% NP-40, 1.2 M sucrose), HaltTMProtease and Phosphatase Inhibitor Single-Use Cocktail (Thermo Fisher Scientific, Inc., Waltham, MA, USA) and centrifuged at 4000 rpm for 10 min at 4 °C. After centrifugation, the supernatant containing the cytoplasmic fraction was transferred to a new tube. The nuclear pellet was resuspended in Nuclear Extraction Buffer (50 mM Tris-HCl pH8.0, 450 mM NaCl, 1.5 mM MgCl_2_, 0.2% NP-40, 10% glycerol) supplemented with HaltTMProtease and Phosphatase Inhibitor Single-Use Cocktail (Thermo Fisher Scientific, Inc., Waltham, MA, USA) and 1mM DTT just prior to use, and incubated with rotation for 30 min at 4 °C. If the lysate was going to be used for immunoprecipitation 1.5:1000 (*v*/*v*) Benzonase^®^Nuclease (Sigma-Aldrich, St. Louis, MO, USA) was added to the Nuclear Extraction Buffer to eliminate nucleic acids. After the incubation, the nuclear lysates were centrifuged at 13,000 rpm at 4 °C for 10 min. The supernatant (soluble nuclear fraction) was transferred to a new tube and the pellet (chromatin and membrane debris) was also stored. To assess protein localisation, lysis buffer was added to the different cellular fractions to equalise the volumes. 

### 2.3. Large-Scale Immunoprecipitation

PC-3 and SKOV-3 cells were lysed in 50 mM Tri-HCl pH 8.0, 150 mM NaCl, 0.1% NP-40, 1 mM EDTA, 2 mM MgCl_2_ and complete™ Mini, EDTA-free Protease Inhibitor Cocktail (Roche, Basel, Switzerland) and incubated with 1.5:1000 (*v*/*v*) Benzonase^®^Nuclease (Sigma-Aldrich, St. Louis, MO, USA) for 30 min at 4 °C to eliminate nucleic acids from the lysates. Total protein was quantified using the Bradford method. In total, 40 μg of HMGB1 antibody (Abcam, ab 18256, Cambridge, UK), THOC5 antibody (Abcam ab86070) or anti-rabbit IgG antibody (Millipore, Co., Burlington, MA, USA) were crosslinked to 50 μL of Protein G-Dynal beads (Invitrogen, Waltham, MA, USA) as previously described [11]. For each IP, 2.5–3 mg of total protein were incubated with antibody-coupled beads for 4 h at 4 °C and beads were then washed four times with IPP150 buffer (10 mM Tris-HCl pH 8.0, 150 mM NaCl and 0.1% NP-40) and four times with 50 mM ammonium bicarbonate. On-bead digestion was carried out as previously described [12]. 

### 2.4. Mass Spectrometry and Data Analysis 

Peptides from large-scale IPs were analysed with online nanoLC-MS/MS on an Orbitrap Velos mass spectrometer coupled with an Ultimate 3000 RSLCnano System. Samples were first loaded and desalted on a nanotrap (100 μm id × 2 cm) (PepMap C18, 5 μ) at 10 μL/min with 0.1% formic acid for 10 min and then separated on an analytical column (75 μm id × 25 cm) (PepMap C18, 2μ) over a 120 min linear gradient of 4–32% CH3CN/0.1% formic acid at 300 nL/min, and the total cycle time was 150 min. The Orbitrap Velos was operated in standard data-dependent acquisition. The survey scans (m/z 380–1500) were acquired in the Orbitrap at a resolution of 30,000 at m/z 400, and one micro-scan was acquired per spectrum. The 10 most abundant multiply charged ions with a minimal intensity of 2000 counts were subject to MS/MS in the linear ion trap at an isolation width of 2 Th. Dynamic exclusion width was set at ±10 ppm for 45 s. The automatic gain control target value was regulated at 1 × 10^6^ for the Orbitrap and 5000 for the ion trap, with maximum injection time at 200 ms for Orbitrap and 100 ms for the ion trap, respectively. The raw files were processed with Proteome Discoverer v1.4 (Thermo Fisher Scientific, Inc., Waltham, MA, USA). Database searches were performed with Mascot (Matrix Science, Inc., Boston, MA, USA) against the human Uniprot database (2014, 77,606 entries) and an in-house contaminant database. The search parameters were trypsin/P digestion, 2 missed cleavages, 10 ppm mass tolerance for MS, 0.5 Da mass tolerance for MS/MS, with variable modifications of acetyl (N-terminal), carbamidomethyl (C), N-formylation (protein), oxidation (M), and pyro-glu (N-term Q). Database search results were refined through processing with Percolator (significance threshold < 0.05, FDR < 1%). Protein identification required at least one high-confidence peptide (FDR < 1%) with a minimum score of 20. External contaminants (albumin, casein, trypsin) were removed from protein lists before further analysis. Keratins were not removed, as they could potentially represent true interactors. To discriminate specific from non-specific interactions, the identified proteins in each IP (specific antibody as experiment and IgG antibody as negative control) were analysed with the Significance Analysis of INTeractome (SAINT) score SAINTexpress [13]. Results from each experiment were analysed using their corresponding negative control. Preys with SAINT probability score cut-off of 0.7 detected by at least two unique peptides were deemed high confidence HMGB1 interacting proteins and further analysed for biological significance. The mass spectrometry proteomics data have been deposited to the ProteomeXchange Consortium via the PRIDE [14] partner repository with the dataset identifier PXD026258. 

### 2.5. Yeast Two-Hybrid Assays 

*HMGB1* and *RBBP7* genes were amplified from commercial clones using the following primers: 

for *HMGB1:*

5′-ATCGAATTCCCGGGGATCGGCAAAGGAGATCCTAAGAAGCC-3′ 

5′-CTCTGCAGGTCGACATCGTTAATCATCATCATCATCTTCTTCTTC-3′; 

for *RBBP7:*

5′-CCGGAATTCCCGGGGATCGCGAGTAAAGAGATGTTTGAAGATAC-3′

5′-CTCTGCAGGTCGACATCGTTAAGATCCTTGTCCCTCCAGTTC-3′. 

Once amplified, the genes were cloned in pGAD-C2 and pGBD-C2 vectors (15), respectively. These vectors allow expression of HMGB1 and RBBP7 proteins fused to the domains Gal4-AD (Activation domain) or Gal4-BD (DNA binding domain) of the yeast transcriptional activator Gal4. The expression of the fused genes is controlled by the *ADH1* gene promoter. For yeast-two hybrid assays both constructs were co-transformed into the two-hybrid host strain PJ69-4A (*MATa trp1-901 leu2-3,112 ura3-52 his3-200 gal4Δ gal80Δ LYS2::GAL1-HIS3 GAL2-ADE2 met2::GAL7-lacZ*) developed by James et al. [15], which allows the use of three reporter genes induced by Gal4 (*HIS3*, *ADE2*, *lacZ*) for assessing protein–protein interactions. Yeast transformants were selected in minimal complete medium (CM) without Trp and Leu and positive candidates were selected after growing them in CM without His and CM without Ade and testing them for β-galactosidase activity. β-galactosidase activity was measured following the method developed by Rose and Botstein [16] using ONPG as substrate. In total, 150 μL of total protein was mixed with 850 μL of Z Buffer (40 mM Na_2_HPO_4_, 60 mM NaH_2_PO_4_, 10 mM KCl, 1 mM MgSO_4_, 50 mM 2-Mercaptoethanol). After incubation at 28 °C for 5 min, 200 μL of 4 mg/mL ONPG in Z buffer were added. Reaction was stopped by addition of 500 μL 1 M Na_2_CO_3_ when a colour change was spotted, and reaction time was written down. Absorbance measures were taken at 420 nm. Specific activity was calculated as (A420 × 1.7)/(0.0045 × protein concentration × extract volume × time). Protein concentration is measured in mg/mL, extract volume in mL and time in minutes. 

### 2.6. Small Scale Immunoprecipitation and Western Blot

PC-3 lysates were obtained as described above. A total of 25 μL of beads were coupled with 5 μg of antibody. Antibodies were not crosslinked to protein G-Dynal beads (Invitrogen). IPs were carried out with Abcam antibodies to THOC5 (ab86070), THOC2 (ab129485), RAB11 (ab3612), and Millipore Normal Rabbit IgG Polyclonal (12–360) was used as the negative control. Then, 1 mg of total protein was used for each IP. Proteins were eluted by incubation in 1× LDS loading buffer (Life Technologies, Carlsbad, CA, USA) containing 350 mM beta-mercaptoethanol at 95 °C for 10 min.

For Western blot, protein samples were run on NuPAGE 4–12% Bis-Tris Protein Gels (Novex, Thermo Fisher Scientific, Inc., Waltham, MA, USA) for 50 min at 200 V and transferred onto a nitrocellulose or PVDF membrane at 30 V for 1 h. Membranes were blocked with PBS-0.1% Tween-20 (PBST) (137 mM NaCl, 2.7 mM KCl, 10 mM Na_2_HPO_4_, 2 mM KH_2_PO_4_, 0.1% Tween-20) containing 5% non-fat milk for 1 h at room temperature or at 4 °C overnight. Membranes were then incubated with the primary antibody: anti-GAPDH (Santa Cruz Biotechnology, Dallas, TX, USA, sc-25778, 1/2000), anti-RBBP4 (Abcam ab1765, 1/4000), anti-Histone3-Lys14acetylated (Millipore, Co., Burlington, MA, USA, 07-353, 1/2000), anti-THOC5 (Abcam, Cambridge, UK, ab86070, 1/1000), anti-THOC2 (Abcam ab129485, 1/1000), anti-SEPT7 (Abcam, Cambridge, UK, ab186021, 1/1000), anti-SEPT2 (Abcam, Cambridge, UK, ab18020, 1/1000) for 1 h at RT or with anti-HMGB1 (Santa Cruz Biotechnology, Dallas, TX, USA, sc-74085, 1/1000) and anti-RAB11 (Abcam, Cambridge, UK, ab3612, 1/1000), at 4 °C overnight. After washing with PBST, membranes were probed with the corresponding horseradish peroxidase-conjugated secondary antibodies: anti-protein G, HRP conjugate (Millipore, Co., Burlington, MA, USA, 18–161, 1/5000) or anti-rabbit HRP (1/2000). After washing the membranes with PBST, chemiluminescence analysis was performed using Amershan-TMECLTM-Prime Western blotting detection reagents according to the manufacturer’s instructions. X-ray films or Bio-Rad ChemiDocTM imager were used for chemiluminescence detection.

### 2.7. HMGB1 Silencing 

siRNAs directed against HMGB1 (siRNA-HMGB1: s20254 Silencer Select) and unspecific control (siRNAControl2: 4390846) were purchased from Ambion Inc. (Thermo Fisher Scientific, Inc., Waltham, MA, USA). Transfection of cells and verification of mRNA and HMGB1 protein downregulation by qRT-PCR and Western blot were done as previously described [9].

### 2.8. qPCR Analysis of Gene Expression

RNA samples from PC-3 and DU-145 cell cultures were obtained using GeneJET RNA Purification Kit (Thermo Fisher Scientific, Inc., Waltham, MA, USA). Reaction conditions for thermal cycling and relative expression calculation were already described [9].

The following primers were used for qPCR amplification. *KRR1*, 5′-CCAAAGAGGACAATCCCAGAG-3′ and 5′-TGTTCATTTAAGGCTTTCTGCAC-3′; *BOP1*, 5′-CGAGATCTGGGAGTGCTGGA-3′ and 5′-CTAGGTGAAGAGGCGA CAGTC-3′; *RRS1*, 5′-TGAACAGCAAGAAGCCTCAGC-3′ and 5′-CCCTTCTGGCT CATTTTCCTC-3′; *LYAR*, 5′-GCAAGGGGAGGTGAAGAAGAA-3′ and 5′-GGCTTCTGATTCCTTGAGTTTTC-3′; *UBTF*, 5′-CTCGGAGGAGAAACGGCG-3′ and 5′-TTCTCAGACAGGTCGTTCCACA-3′.

## 3. Results

### 3.1. HMGB1 Interactome in Ovary and Prostate Cancer Cell Lines 

**Figure 1:**
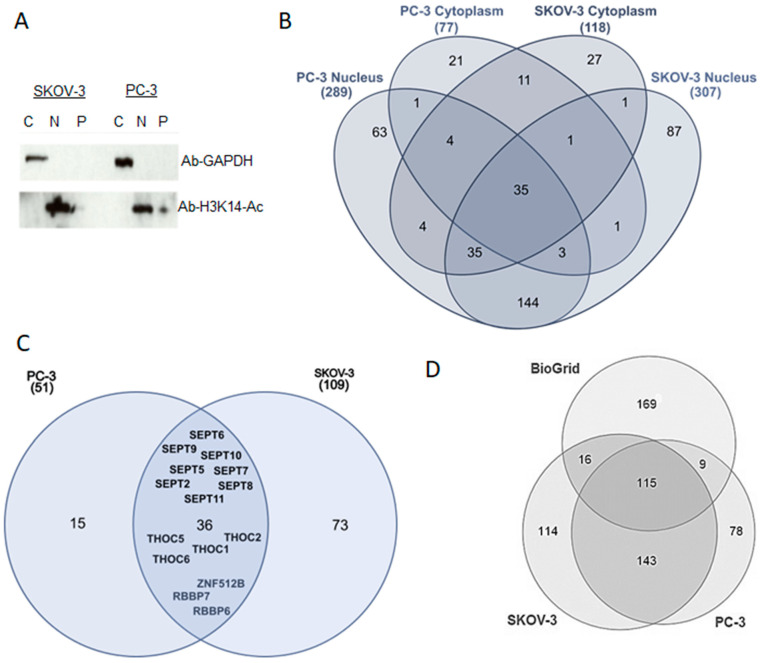
HMGB1 interactome. (**A**) Western blot verification of cellular fractionation of SKOV-3 and PC-3 lysates: nuclear –N- and cytoplasmic –C- fractions and residual pellet –P- were separated in polyacrylamide gels and hybridised against GADPH antibody or H3K14-Ac antibody. (**B**) Venn diagram that summarises the distribution of HMGB1 interactions detected in nuclear and cytoplasmic fractions of SKOV-3 and PC-3 cell lines. (**C**) Venn diagram remarking common interactions detected in SKOV-3 and PC-3 cells. (**D**) Venn diagram showing the intersection of proteins detected in the HMGB1-interactomes with other previously reported HMGB1 binding proteins (https://thebiogrid.org/, accessed on 20 February 2021).

To increase our understanding of HMGB1 cellular functions in cancer cells, we profiled HMGB1 interactomes in SKOV-3 and PC-3 cancer lines from ovarian and prostate tumours, respectively, by affinity purification followed by mass spectrometry (AP-MS). We carried out three independent HMGB1 immunoprecipitation experiments and corresponding isotype IgG controls from whole cell extracts of each cell line, which identified 109 and 50 HMGB1 interacting proteins in SKOV-3 and PC-3 cells, respectively (Tables S1 and S2). In addition, we also performed one replicate AP-MS experiment from nuclear and cytoplasmic extracts separately in each cell line with their corresponding IgG controls. Subcellular fractionation was verified by Western blot (Figure 1A and Figure S3). This allowed us not only to map interactions to the nuclear and cytoplasmic compartments, but also enabled the detection of a higher number of HMGB1 interacting partners to those detected in whole cell lysates. The majority of interactions were identified in the nuclear fraction, in agreement with HMGB1′s main cellular localisation [17]. We identified 306 and 289 interactions in the nuclear fractions of SKOV-3 and PC-3 cells respectively (Tables S3 and S4), compared to 117 and 76 interactions (Tables S5 and S6) in the corresponding cytoplasmic fractions (Figure 1B). Over 50% of identified proteins were common to PC-3 and SKOV-3 cells (Figure 1C). Comparison with the BioGrid interaction database (https://thebiogrid.org, accessed on 20 February 2021) revealed that 140 out of 438 total interactions (32%) identified in our study had already been reported in mammalian cells (Figure 1D), indicating that our AP-MS approach is able to capture known HMGB1 interactions and reinforcing the reliability of the novel interactions discovered in our study.

GO-term enrichment analyses were carried out on HMGB1 associated proteins detected in whole-cell lysates (Table 1), as well as nuclear or cytoplasmic fractions of SKOV-3 and PC-3 cells (Table 2 and Figure S1). As expected from previous reports on HMGB1 functions (5, 10), in the nuclear interactome we observed an enrichment of proteins associated with the GO-terms chromatin organisation, nucleosome organisation, epigenetic regulation of gene expression and DNA repair. In addition, an important fraction of proteins detected in the nuclear HMGB1 interactome are functionally connected to RNA processing of different rRNAs, mRNA splicing via spliceosome, ribonucleoprotein (RNP) complex biogenesis and RNP export from nucleus. In the cytoplasmic interactome, we observed enrichment of the GO-terms translation, SRP-dependent co-translational protein targeting to membrane, regulation of translation, and cell division.

**Figure 2:**
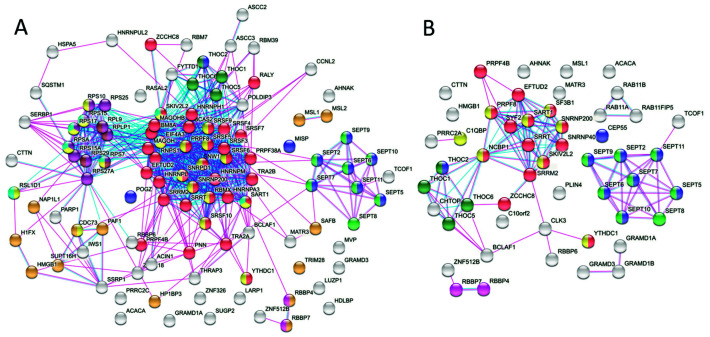
Interaction networks of HMGB1-associated proteins in SKOV-3 and PC-3 cell lysates showing principal clusters. Colour code is as follows: red, mRNA splicing, via spliceosome (GO:0000398); dark blue, rRNA processing (GO:0006364); green, ribonucleo-protein complex biogenesis (GO:0022613); yellow, nuclear transport (GO:0051169); pink, chromatin organisation (GO:0006325); purple cell division (GO:0051301); brown, DNA repair (GO:0006281); dark green, NuRD complex (GO:0016581).

Interaction network analysis of the HMGB1 interactomes obtained from SKOV-3 and PC-3 cell lysates with STRING [18] revealed three interesting clusters that corresponded to components of the NuRD and THOC nuclear complexes, as well as the septin complex (Figure 2).

### 3.2. HMGB1 Interacts with Nucleosome Remodelling (NuRD) Complex Subunit RBBP7

**Figure 3:**
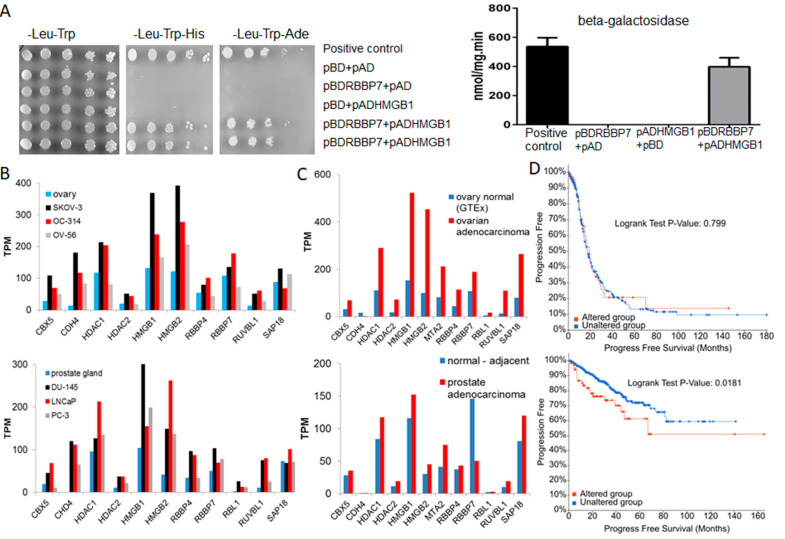
(**A**) Validation of the HMGB1-RBBP7 interaction by the two-hybrid approach. (**B**) Differential mRNA levels of NuRD complex subunits in normal and cancerous ovary and prostate cell lines. (**C**) Differential mRNA levels of NuRD complex subunits in tumours and adjacent normal tissue in patients diagnosed of ovary and prostate cancers. (**D**) Analysis of disease-free progression in patients with mRNA levels of NuRD subunits higher (altered group) or lower (unaltered group) than mean + 2SD. In (**B**–**D**) the upper part is from ovary cancer and lower part from prostate cancer. The source of data used in the analyses B-D is detailed in the text.

We repeatedly identified several subunits (MTA2, CHD4, RBBP4, HDAC1, RBBP7, HDAC2) of the NuRD complex in HMGB1 IP experiments in both whole cell lysates and nuclear fractions from SKOV-3 and PC-3 cells (Tables S1–S4). CHD4, MTA2, HDAC1/2 were only detected in nuclear interactomes, whilst RBBP4 and RBBP7 were detected in both whole cell and nuclear interactomes, together with ZNF512B, which is a substoichiometric interactor of the NuRD complex [19]. CHD4 was only detected in the SKOV-3 cell line. The NuRD complex has been associated to transcriptional processes, chromatin assembly mechanisms, cell cycle progression, genomic stability and epigenetic control of gene expression [20]. Despite being involved in the same processes, no previous interactions between HMGB1 and NuRD complex subunits have been reported. To validate this association, we tested for a direct physical interaction using the yeast two-hybrid assay [15]. We selected for this assay two candidates in the complex, RBBP4 and RBBP7, dynamic core subunits of the NuRD complex, which act as histone chaperones [19]. We confirmed a direct physical association between HMGB1 and RBBP7 (Figure 3A), demonstrating that these proteins do indeed interact. We did not detect an interaction with RBBP4 (data not shown), suggesting that RBBP4 might be linked to HMGB1 through RBBP7.

Since HMGB1 overexpression has been associated to cancer in different cell types [2,3,4] we looked for evidence of overexpression of the NuRD complex in cancerous cells. Data available through Expression Atlas, Cancer Cell Line Encyclopedia (Figure 3B) showed higher mean values of mRNA expression levels of components of the NuRD complex in different cancer cell lines (including SKOV-3 and PC-3) than in the corresponding non-cancer cells from ovary (https://www.ebi.ac.uk/gxa/experiments/E-MTAB-5214/Results, accessed on 3 March 2021 and prostate (https://www.ebi.ac.uk/gxa/experiments/E-MTAB-3358/Results, accessed on 3 March 2021)

Data from the Pan-Cancer Analysis of Whole Genomes (PCAWG) project also showed (Figure 3C) higher mean values of mRNA expression in tumours than in adjacent normal tissue from patients (https://www.ebi.ac.uk/gxa/experiments/E-MTAB-5200/Results, accessed on 3 March 2021). Concomitantly, using the tools and statistics available through cBioportal (http://www.cbioportal.org/, accessed on 3 March 2021) we found a positive correlation between high levels of expression of five components of the NuRD complex (RBBP4, RBBP7, HDAC1, HDAC2 and MTA2) and a shorter progression free survival, with a logrank test *p*-value of 0.018, in the prostate adenocarcinoma (acinar type) study (TCGA PanCancer Atlas), integrated by 494 patients (Figure 3D). We did not, however, observe a statistically significant effect in the ovarian serous cystadenocarcinoma study (TCGA PanCancer Atlas), integrated by 585 patients (Figure 3D).

### 3.3. HMGB1-Associated THOC and SEPTIN Complexes Interact between Them and with RAB11

**Figure 4:**
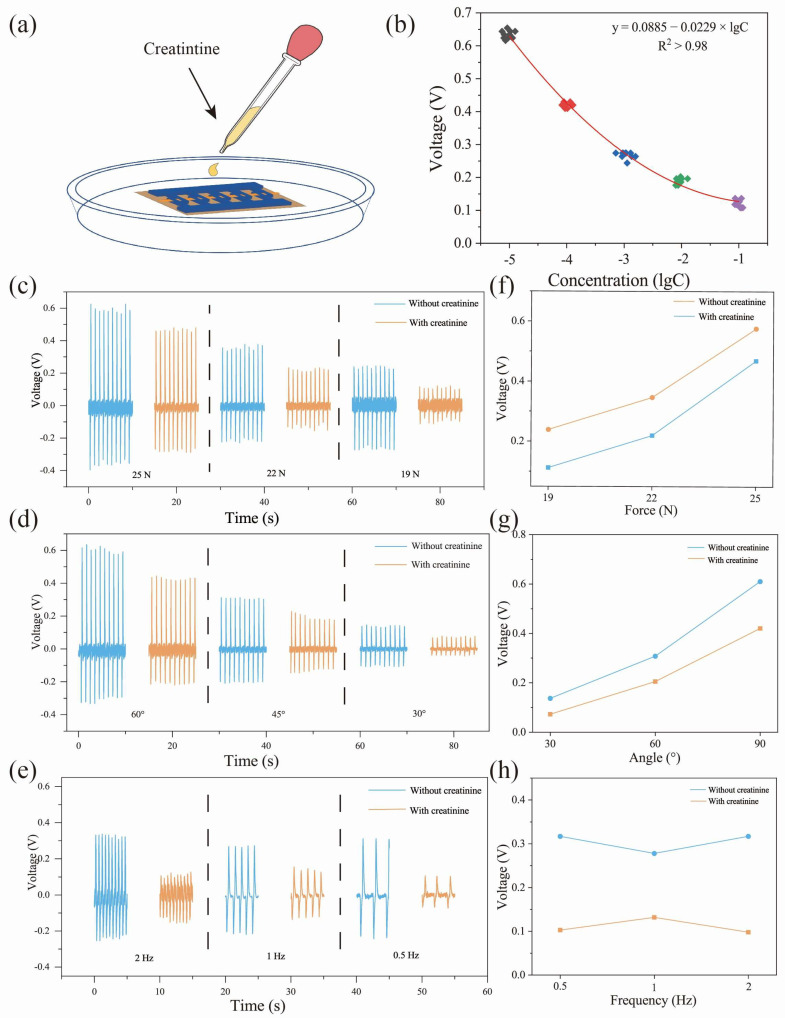
(**a**) Measurement process. (**b**) Relationship between creatinine concentration and pie-zoelectric output voltage. Sensing performance against different applied forces (**c**), bending angles (**d**), and force frequencies (**e**). Comparison of piezoelectric output variation trends under different force sizes (**f**), bending angle (**g**), and force frequencies (**h**) before and after adding creatinine.

In both HMGB1 prostate and ovarian cytoplasmic and nuclear interactomes we detected an enrichment of the septin complex, composed of SEPT6, SEPT9, SEPT7, SEPT11, SEPT10, SEPT8, and SEPT2 (Figure 2). In the course of our study a physical interaction between SEPT9 and HMGB1 in cervix cancer was reported [21] further validating our results. Septins are most commonly known as cytoskeleton GTP binding proteins [22] but have recently also been reported as nuclear proteins involved in RNA processing [23]. We also noticed an enrichment of the THOC complex (THOC1, THOC6, THOC5, THOC2) in the nuclear interactomes. THOC is a subcomplex of the “transcription/export” complex (TREX complex) [24] and its structure in humans has recently been resolved by cryo-electron microscopy, revealing a hub for multivalent interactions [25]. This raised the question of whether these two complexes interact. In order to explore this hypothesis, we carried out THOC5 immunoprecipitation from PC-3 cells, followed by MS analysis. In total, 282 proteins were identified as confident THOC5 interacting proteins (Table S7), several of which we had identified also as HMGB1 binding partners (Figure 4A). Among THOC5 interacting partners we detected other THOC proteins (THOC1, THOC2, THOC3, THOC6 and THOC7) as expected, as well as septin proteins (SEPT2, SEPT7, SEPT9 and SEPT11). In order to verify these interactions, we carried out small scale immunoprecipitations from PC-3 cell lysates followed by Western blotting with antibodies against THOC5, THOC2, SEPT2, SEPT7 (Figure 4B). These experiments confirmed the physical interactions between THOC5 and THOC2, THOC2 and SEPT2, and THOC2 and SEPT7. We did not detect HMGB1 in THOC or SEPT immunoprecipitations; this could be due to HMGB1 representing only a minor undetectable fraction of proteins interacting with the septin and THOC complexes, or antibody-mediated displacement issues. We also probed the blots with an antibody against the small GTPase RAB11, since this protein was identified in both HMGB1 and THOC5 interactomes in prostate cancer cells (Figure 4A, Tables S2 and S4). A band corresponding to RAB11 was clearly detected in THOC2 and THOC5 immunoprecipitates. In addition, we could also detect SEPT2 and SEPT7 in RAB11 immunoprecipitates (Figure 4B and Figure S3). These results indicate that the septin and THOC complexes interact between them and with RAB11.

### 3.4. Clinical Significance of Expression Changes of HMGB1 Interactome Components Related to RNA Processing and Nuclear Export in Prostate and Ovary Cancers

**Figure 5:**
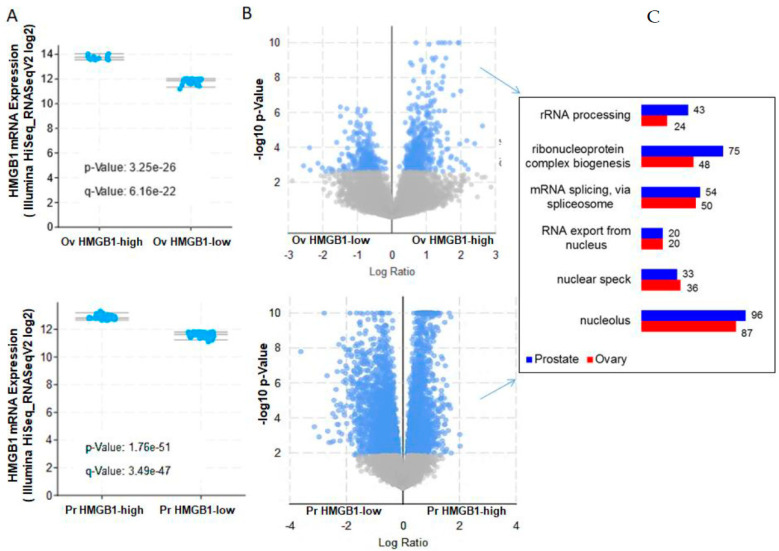
Differential expression of genes related to RNA processing in tumours expressing HMGB1 at high or low levels. (**A**) Selection of samples expressing mRNA HMGB1 at levels > median + 1.5 SD (HMGB1-high) and samples expressing mRNA HMGB1 at levels < median − 1.5 SD (HMGB1-low). (**B**) Volcano plots of genes expressing at higher levels in each group; log ratio in HMGB1-high/HMGB1-low. Ov, samples obtained from the PanCancer ovarian cystadenocarcinoma ATCG study in cBioportal; Pr, samples obtained from the PanCancer prostate adenocarcinoma ATCG study in cBioportal. (**C**) GO term enrichment analysis of genes expressed at higher levels in HMGB1-high samples in ovary or prostate cancer.

We next analysed whether mRNA expression levels of HMGB1 and its interactome partners in ovary and prostate tumours are correlated. To perform these analyses, we used the tools and statistics of cBioportal (https://www.cbioportal.org, accessed on 3 March 2021) and defined two groups of samples in the prostate and ovary cancer studies of the TCGA Pan Cancer Atlas; one group composed of samples with *HMGB1* mRNA levels lower than mean − 1.5 standard deviation (HMGB1-low) and a second group expressing mRNA levels higher than mean + 1.5 standard deviation (HMGB1-high) (Figure 5A). After differential gene expression analysis between the two groups, we found 31 and 79 genes (in prostate adenocarcinoma and ovary cystadenocarcinoma, respectively) encoding HMGB1-interacting proteins, which were more highly expressed in the HMGB1-high group with statistical significance according to the cBioportal analysis record (Figure 5B). GO term enrichment analysis of genes expressed at higher levels in the tumours classified as HMGB1-high (Figure 5C) showed that these include the functional terms rRNA processing, RNP biogenesis, mRNA splicing, and RNA export from nucleus to cytoplasm. In agreement with these functions, the cellular compartment GO terms nucleolus and nuclear speckles were also enriched. Indeed, we noticed that both the nuclear and cytoplasmic HMGB1 interactomes contained a high proportion of proteins previously described as part of the RNA interactome in Hela cells (26) and that many nuclear HMGB1-interacting proteins (119 in SKOV-3 and 100 in PC-3 cells) were related to RNA processing. HMGB1 has traditionally been considered as a non-histone chromatin protein. Therefore, we explored in more detail their nuclear distribution using cellular component GO-term annotations (Table 3). We confirmed that although approximately 25% are associated to chromatin structural and modifier components, a higher proportion, 32–39%, is associated to nucleolus, where rRNA transcription and processing occurs, as well as to spliceosome (14–22%), and mostly to catalytic step 2 of the spliceosome mRNA processing pathway during intron elimination. Approximately 14–20% are associated to nuclear speckles and 6–7% are associated to nuclear membranes. Among KEGG pathways identified, we found hsa03040 (Spliceosome), hsa03008 (Ribosome biogenesis in eukaryotes), hsa03013 (RNA transport). 

**Figure 6:**
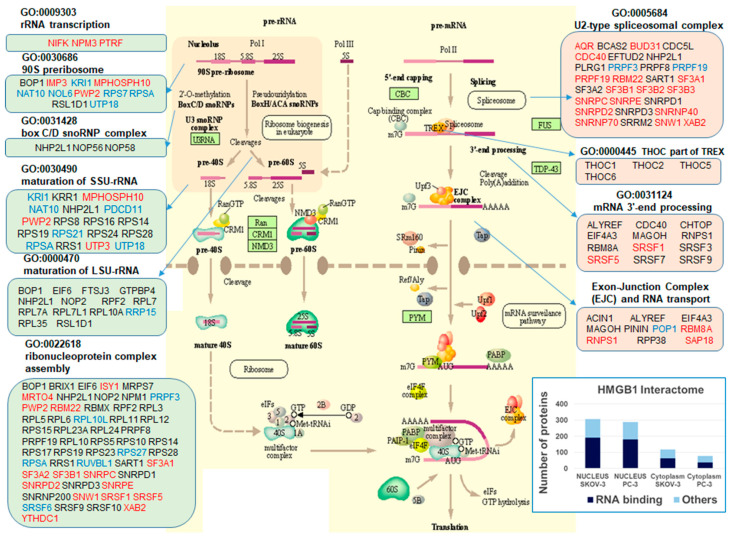
Mapping of HMGB1 interactome to the RNA processing and export pathways (adapted from the KEGG map RNA TRANSPORT from Kanehisa laboratories hsa03013, accessed 22 July 2020). Red, proteins found in SKOV-3; blue, in PC-3; black, in both cell lines. The graph in the bottom right box shows the total number proteins identified and those common to the RNA interactome described in HELA cells [26].

A detailed distribution of selected proteins from the nuclear interactomes of SKOV-3 and PC-3 cells involved in the RNA processing and export pathway is shown in Figure 6. These results suggest that HMGB1 could have an as yet unreported role in ribosome biogenesis and/or splicing. To explore the biological relevance of this observation, we obtained the list of genes that are synthetic lethal with HMGB1 from DepMap (https://depmap.org, accessed on 19 July 2021). The top 100 HGMB1-dependent CRISPR gene set was enriched in genes involved in ribosome biogenesis (adj. *p*-value 1.82 × 10^−2^), rRNA processing (adj. *p*-value 1.87 × 10^−2^) and mRNA transport (adj. *p*-value 2.59 × 10^−2^), and genes coding for RNA-binding proteins (adj. *p*-value 3.11 × 10^−7^), further reinforcing a role for HMGB1 in these processes.

**Figure 7:**
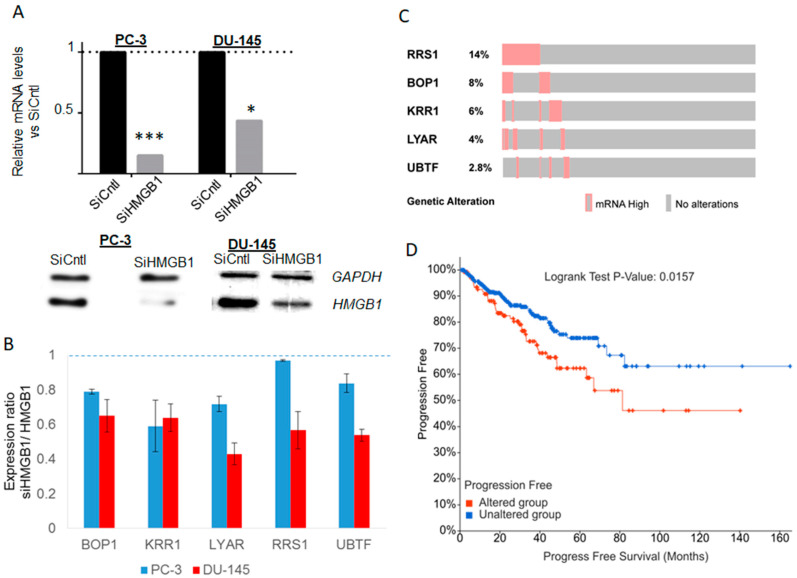
Effects of HMGB1 silencing on gene expression. (**A**) Verification of HMGB1 silencing by qPCR. (up) and Western blot (down); * *p* < 0.05, *** *p* < 0.001 (**B**) Effects of HMGB1 silencing on the expression of genes with regulatory functions in RNA processing and/or ribosome biogenesis. (**C**) Percentage of adenocarcinoma tumours with high expression (mean+2SD) of genes encoding regulators of rRNA synthesis and ribosome biogenesis. (**D**) Correlation of the expression of genes encoding these regulators (SRR1, BOP1, KRR1, LYAR, UBTF) and progression-free periods in prostate adenocarcinoma patients.

We also analysed which genes encoding the proteins found in the HMGB1 interactomes are more frequently highly expressed in tumour samples from prostate adenocarcinomas, and we found among them *RRS1* and *BOP1*, both important regulators of ribosome biogenesis. In order to assess how HMGB1 expression affects these genes, the *HMGB1* gene was silenced by iRNA approach as previously described (8) in the prostate cell lines PC-3 and DU-145 (Figure 7A). Levels of expression of *RRS1*, *BOP1*, as well as *KRR1*, *LYAR* and *UBTF*, also implicated in the control of ribosome biogenesis and found in our interactome study, were measured in silenced and control cells (Figure 7A). Results showed that expression levels of all candidates except *KRR1* were lower in one or both cell lines when HMGB1 was silenced (Figure 7B). A percentage of adenocarcinoma tumours show high expression of these ribosome biogenesis genes (Figure 7C). In addition, higher expression of these genes was associated to shorter progression-free survival (logrank test *p*-value 1.57 × 10^−2^) in patients (Figure 7D). To explore whether a correlation exists between the expression of genes related to RNA processing/RNA nuclear export, and clinical outcome of patients with prostate or ovary tumours, we also analysed survival data available in TCGA Pan Cancer Atlas through cBioportal. In the prostate adenocarcinoma study we found that higher expression of these genes was positively correlated with worse progression-free survival for LSU-rRNA (logrank test *p*-value 7.11 × 10^−3^), 5.8S rRNA from tricistronic rRNA transcript (logrank test *p*-value 6.19 × 10^−3^), RPN complex assembly (logrank test *p*-value 5.73 × 10^−3^), mRNA splicing at catalytic step 2 (logrank test *p*-value 5.51 × 10^−3^), and nucleo-cytoplasmic transport (logrank test *p*-value 1.08 × 10^−3^) related genes (Figure S2). This correlation was not found in ovary cystadenocarcinoma.

Together, these data suggest that HMGB1 has a role in ribosome biogenesis through interaction with and regulation of expression of genes involved in this process, with significant clinical impact in cancer patients.

## 4. Discussion

The functions associated to proteins interacting with HMGB1 in nucleus and cytoplasm reported in our study provide new clues shedding light on the contribution of HMGB1 to prostate and ovary oncogenesis. The identification of common interactors of HMGB1 in PC-3 and SKOV-3 cells lines is noteworthy and might suggest that the oncogenic role of HMGB1 overexpression affects basic cellular functions that are altered in different cancers. Previous data on HGMB1 functions pointed to its involvement in DNA related processes, like replication, transcription and DNA repair, as well as in apoptosis/autophagy balance and extracellular signalling [5]. Our interactome investigation has revealed novel HMGB1 binding partners which strongly associate HMGB1 function with RNA metabolic processes, such as rRNA transcription and processing, mRNA processing in the spliceosome, RNA and RNP transport from nucleus to cytoplasm and ribosome assembly. These processes have great importance in cell proliferation, differentiation, cell survival, and tumorigenesis [27], and we have found a positive correlation between higher expression of HMGB1 interacting proteins with worse patient prognosis in prostate cancer. Since cancer development demands a higher rate of cell division and metabolism, which requires an increase in protein synthesis, alterations in ribosomal proteins networks necessarily play a role in tumour development. Importantly, other HMGB proteins of different origins from yeast to human have previously been associated with ribosome biogenesis [28].

A significant number of proteins characterised in our interactome, including HMGB1, were previously identified as RNA-binding proteins in cancer cells [26]. This is remarkable since some RNA-binding proteins are also known as post-transcriptional drivers of cancer progression [29]. This characteristic, together with their functions in the RNA processing and ribosome biogenesis pathways suggest that HMGB1 might connect the transcription of specific pre-rRNAs and pre-mRNAs to their processing, nucleo-cytoplasmic export and final participation in translation, via protein–protein interactions. 

Supporting this hypothesis, we found a positive correlation between increased *HMGB1* gene expression and increased expression of genes involved in these processes, both in ovary cystadenocarcinoma and prostate adenocarcinoma tumours. In addition, we verified that HMGB1 silencing causes a downregulation of genes participating in rRNA processing and ribosome biogenesis (*RRS1*, *BOP1*, as well as of *KRR1*, *LYAR* and *UBTF* in PC-3 and/or DU-145 cells). *RSS1* controls ribosome biogenesis through the 5S RNP complex, composed of rRNA 5S, RPL5 and RPL11 (proteins also identified in the present HMGB1 interactome) and is responsible for their nucleolar localisation [30]. It has been reported that higher levels of *RSS1* and *BOP1* are observed in 14% and 8%, respectively, of prostate adenocarcinomas and that higher levels of *RRS1* mRNA correlate with shorter progression-free periods. *BOP1* knockdown results in decreased proliferation and motility [31], implicating this gene in prostate cancer aggressiveness. *UBTF* is a HMGB-box factor whose depletion causes apoptotic death in transformed cells [32] and is involved in RNA Pol I transcription and regulation of highly transcribed RNA Pol II genes [33]. *KRR1* is related to pre-40S subunit assembly during ribosome biogenesis [34], and *LYAR* is involved in formation of pre-rRNAs and their subsequent processing to produce 18S and 28S rRNAs [35] and pre-ribosome assembly [36]. 

We also detected an interaction of HMGB1 with components of several protein complexes related to rRNA synthesis and ribosome biogenesis, namely NuRD, THOC and septin complexes.

We show that in the nucleus HMGB1 interacts with several subunits of the NuRD complex prominently in nuclear fractions, and we confirmed its direct physical interaction with RBBP7. In growth-arrested and differentiated cells the NuRD complex is strongly associated to rRNA genes (rDNA) and maintains them in a silent state characterised by methylation and heterochromatic features [37]. The discovered interaction between HMGB1 and RBBP7 could be a factor modifying the silenced state and increasing rRNA synthesis. 

THOC1, THOC2, THOC5 and THOC6, members of the THO subcomplex of TREX [38] which participates in mRNA processing and transport of RNPs from nucleus to cytoplasm, are present in HMGB1 immunoprecipitates from ovary and prostate cancer cells. THOC1 has previously been associated to prostate cancer aggressiveness [39]. THOC5 is highly specialised in the processing of mRNAs related to cancer proliferation, since it contributes to more than 90% of the 3′ processing and/or export of immediate-early genes induced by growth factors and/or cytokines, but only to less than 1% of total mRNA export in the steady state [40,41]. This reinforces our hypothesis that HMGB1 interactions orchestrate the transcription of specific pre-rRNAs and pre-mRNAs to their processing, nucleo-cytoplasmic export and final participation in translation.

Septins, a family of GTP-binding proteins which participate in a spectrum of cellular processes including cytokinesis, cilliogenesis, cell migration, polarity, and cell–pathogen interactions [22,42] were also identified in the HMGB1 interactomes. Surprisingly, in a previous interactome analysis of septins [23], the most significant connections were found with RNA splicing and processing, an unprecedented observation despite extensive prior work on septins. Since both the THOC complex [43] and several septins [23] have been related to nuclear speckles, we decided to look for an interaction between components of the THOC complex and septins in prostate cancerous cells. In the IP-MS experiments carried using THOC5 antibody we identified other components of the THOC complex as well as SEPT2, SEPT7, SEPT9 and SEPT11. Cross validation in PC-3 cell lysates by co-immunoprecipitation confirmed the physical interactions between THOC5 and THOC2, THOC2 and SEPT2, THOC2 and SEPT7. The small GTPase, RAB11, another HMGB1-interacting protein from our IP-MS experiments, also immunoprecipitated with HMGB1 and THOC5. All these data reinforce the function of septins in RNA splicing and processing as well as the involvement of HMGB1 in these functions. It is worth remarking that septins have been linked to cancer, specifically SEPT9 to prostate cancer [44]. In the course of our study a direct interaction between SEPT9 and HMGB1 in cervix cancer was reported [21] further validating our results.

## 5. Conclusions

In conclusion, the analysis of the HMGB1 interactomes in prostate and ovary cancer cells reveals new connections of HMGB1 functions to RNA processing and ribosome biogenesis through interactions with components of the NuRD complex, THOC complex and septins. Given the relevance of all these factors in cancer development and progression, the interaction networks provide new insight on their role in oncogenesis.

## Figures and Tables

**Table 1:** GO term enrichment analysis of the whole lysate in HMGB1-interactomes in SKOV-3 and PC-3 cells. (OGC, observed gene count; BCG, background gene count; FDR, false discovery rate; ns, not statistically significant).

		SKOV-3			PC-3		
ID	Description	OGC	BGC	FDR	OGC	BGC	FDR
GO:0000445	THO complex part of TREX	4	6	2.04 × 10^−6^	4	6	2.64 × 10^−7^
GO:0006396	RNA processing	54	825	6.15 × 10^−41^			ns
GO:0000398	mRNA splicing, via spliceosome	35	284	1.91 × 10^−33^	14	284	6.00 × 10^−12^
GO:0006364	rRNA processing	8	192	0.00024			ns
GO:1990904	Ribonucleoprotein complex	39	770	5.65 × 10^−25^	13	770	8.23 × 10^−7^
GO:0022613	RNP complex biogenesis	20	409	8.51 × 10^−12^	8	409	0.00038
GO:0000387	Spliceosomal snRNP assembly	4	37	0.0010			ns
GO:0071426	RNP complex export from nucleus	20	124	9.01 × 10^−21^			ns
GO:0006325	Chromatin organisation	13	683	0.0016			ns
GO:0034728	Nucleosome organisation	7	167	0.00074			ns
GO:0040029	Regulation of gene expression, epigenetic			ns	6	251	0.0016
GO:0016581	NuRD complex	2	14	0.0187	2	14	4.00 × 10^−3^
GO:0031105	Septin complex	4	6	2.04 × 10^−6^	4	6	2.64 × 10^−7^
GO:0051301	Cell division	10	483	0.0049	9	483	1.7 × 10^−4^
GO:0006614	SRP-dependent cotranslational protein targeting to membrane	11	92	3.44 × 10^−10^			ns
GO:0002181	Cytoplasmic translation	4	57	0.0044			ns
GO:0006417	Regulation of translation	7	327	0.0258			ns
IPR016491	Septin	8	13	1.03 × 10^−11^	8	13	3.40 × 10^−14^

**Table 2:** GO term enrichment analysis of the nuclear and cytoplasmic HMGB1-interactomes in SKOV-3 and PC-3 cells. (OGC, observed gene count; BCG, background gene count; FDR, false discovery rate).

		SKOV-3			PC-3		
ID	Description	OGC	BGC	FDR	OGC	BGC	FDR
NUCLEUS							
GO:0006396	RNA processing	119	825	6.10 × 10^−75^	100	825	3.72 × 10^−59^
GO:0000398	mRNA splicing, via spliceosome	65	284	7.83 × 10^−50^	41	284	1.94 × 10^−25^
GO:0006364	rRNA processing	43	192	3.72 × 10^−32^	51	192	3.54 × 10^−43^
GO:0000470	Maturation of LSU-rRNA	12	24	2.69 × 10^−12^	14	24	1.87 × 10^−15^
GO:0030490	Maturation of SSU-rRNA	12	52	4.08 × 10^−9^	15	52	5.52 × 10^−13^
GO:0000460	Maturation of 5.8S rRNA	4	32	0.0196	9	32	1.21 × 10^−6^
GO:1990904	Ribonucleoprotein complex	149	770	9.45 × 10^−115^	141	770	2.47 × 10^−110^
GO:0022613	RNP complex biogenesis	81	409	1.37 × 10^−58^	79	409	3.94 × 10^−59^
GO:0000387	Spliceosomal snRNP assembly	10	37	3.27 × 10^−8^	7	37	3.70 × 10^−5^
GO:0051169	Nuclear transport	28	267	3.38 × 10^−13^	27	267	3.59 × 10^−13^
GO:0071426	RNP complex nuclear export	18	124	1.37 × 10^−10^	17	124	3.49 × 10^−10^
GO:0006325	Chromatin organisation	42	683	2.47 × 10^−12^	40	683	3.67 × 10^−12^
GO:0034728	Nucleosome organisation	22	167	3.66 × 10^−12^	22	167	3.66 × 10^−12^
GO:0040029	Regulation of gene expression, epigenetic	21	251	2.45 × 10^−8^	17	251	4.77 × 10^−6^
GO:0016581	NuRD complex	6	14	3.05 × 10^−6^	5	14	4.20 × 10^−5^
GO:0031105	Septin complex	4	6	6.96 × 10^−5^	4	6	5.79 × 10^−5^
GO:0006281	DNA repair	19	491	0.0039	15	491	0.0440
GO:0051301	Cell division	21	483	0.00036	24	483	4.47 × 10^−6^
CYTOPLASM							
GO:0006614	SRP-dependent cotranslational protein targeting to membrane	60	92	2.15 × 10^−96^	29	92	2.53 × 10^−43^
GO:0002181	Cytoplasmic translation	27	57	6.52 × 10^−39^	13	57	1.72 × 10^−17^
GO:0006417	Regulation of translation	12	327	1.39 × 10^−5^	11	327	1.31 × 10^−6^
GO:0051301	Cell division	11	483	0.0025	12	483	6.70 × 10^−6^
IPR016491	Septin	8	13	4.74 × 10^−11^	8	13	1.06 × 10^−12^

**Table 3:** GO-term enrichment analysis of the nuclear compartment distribution of HMGB1-nuclear interactomes in SKOV-3 and PC-3 cells. (OGC, observed gene count; BCG, background gene count; FDR, false discovery rate).

		SKOV-3			PC-3		
ID	Description	OGC	BGC	FDR	OGC	BGC	FDR
GO:0005634	Nucleus	243	6892	7.34 × 10^−55^	226	6892	1.96 × 10^−51^
GO:0005654	Nucleoplasm	192	3446	5.34 × 10^−66^	165	3446	1.45 × 10^−50^
GO:0005730	Nucleolus	79	926	2.50 × 10^−33^	88	926	9.18 × 10^−44^
GO:0016604	Nuclear body	63	742	5.02 × 10^−26^	47	742	8.81 × 10^−16^
GO:0005694	Chromosome	60	950	1.13 × 10^−18^	60	950	2.48 × 10^−20^
GO:0005681	Spliceosomal complex	53	187	1.13 × 10^−44^	33	187	6.24 × 10^−23^
GO:0016607	Nuclear speck	47	381	2.12 × 10^−25^	32	381	9.22 × 10^−14^
GO:0071013	Catalytic step 2 spliceosome	43	99	1.68 × 10^−42^	27	99	5.63 × 10^−23^
GO:0000785	Chromatin	35	489	3.15 × 10^−12^	36	489	7.64 × 10^−14^
GO:0000790	Nuclear chromatin	25	333	2.75 × 10^−9^	24	333	4.31 × 10^−9^
GO:0000786	Nucleosome	17	106	7.01 × 10^−11^	15	106	2.51 × 10^−9^
GO:0005635	Nuclear envelope	16	446	0.0079	15	446	0.0118
GO:0000118	Histone deacetylase complex	8	60	6.32 × 10^−5^	7	60	0.00028
GO:0016581	NuRD complex	6	14	3.05 × 10^−6^	5	14	4.20 × 10^−5^
GO:0031105	Septin complex	4	6	6.96 × 10^−5^	4	6	5.79 × 10^−5^

## Data Availability

Proteomic data presented in this study are openly available in ProteomeXchange Consortium via the PRIDE (https://www.ebi.ac.uk/pride/, submitted 21 May 2021) partner repository with the dataset identifier PXD026258.

## Supplementary Materials

The following are available online at https://www.mdpi.com/article/10.3390/cancers13184686/s1, Figure S1: Clustering of proteins that interact with HMGB1 in SKOV-3 and PC-3 cells in nuclear and cytoplasmic fractions, Figure S2: Correlation between high expression of functional groups of genes related to the PC-3 HMGB1 interactome and progression free periods in prostate adenocarcinoma patients, Figure S3: Original Western Blot., Table S1: HMGB1 interactome in SKOV-3 cells, Table S2: HMGB1 interactome in PC-3 cells, Table S3: HMGB1 interactome in the nuclear fraction of SKOV-3 cells, Table S4: HMGB1 interactome in the nuclear fraction of PC-3 cells, Table S5: HMGB1 interactome in the cytoplasm fraction of SKOV-3 cells, Table S6: HMGB1 interactome in the cytoplasm fraction of PC-3 cells, Table S7: THOC5 interactome in PC-3 cells.
