# Supplementary material for: HMGB1 Protein Interactions in Prostate and Ovary Cancer Models Reveal Links to RNA Processing and Ribosome Biogenesis through NuRD, THOC and Septin Complexes
Source: Cancers (Basel). 2021 Sep 18;13(18):4686. doi: 10.3390/cancers13184686 (PMC8466577; doi:10.3390/cancers13184686)
Supplement: Supplementary file 1 [file cancers-13-04686-s001.zip › cancers-1365269 supplementary figure revised by authors.pdf]

cancers

Supplementary Material: HMGB1 Protein Interactions in Prostate and Ovary Cancer Models Reveal Links to RNA Processing and Ribosome Biogenesis through NuRD, THOC and Septin Complexes

Aida Barreiro-Alonso, Mónica Lamas-Maceiras, Lidia Lorenzo-Catoira, Mercedes Pardo, Lu Yu, Jyoti S. Choudhary and M. Esperanza Cerdán

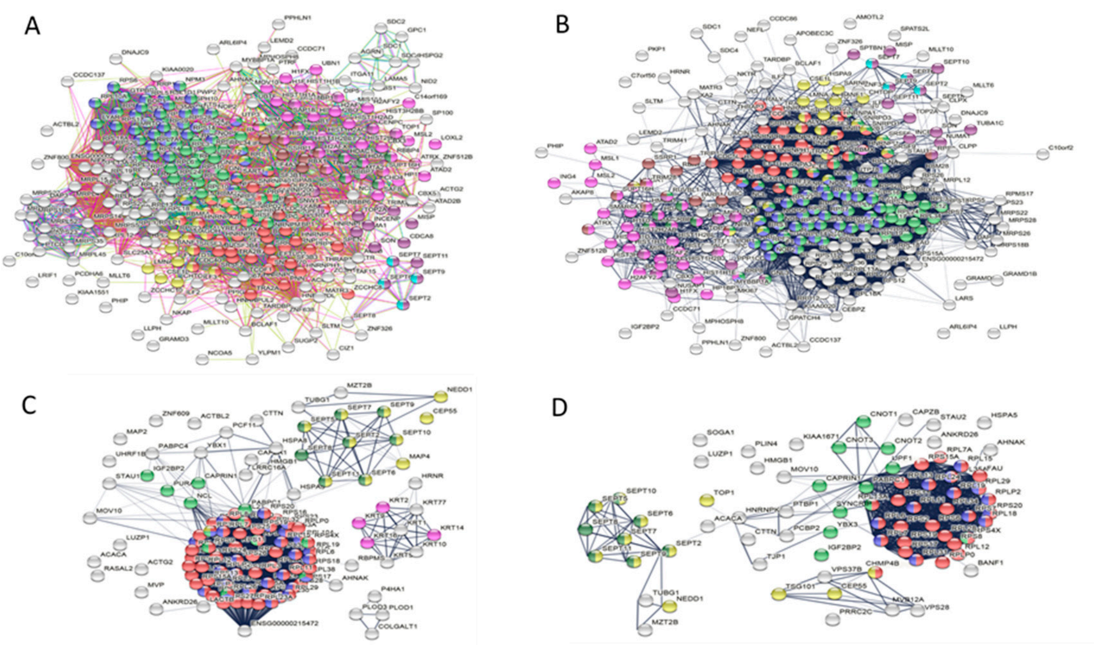

Copyright: © 2021 by the authors. Licensee MDPI, Basel, Switzerland. This article is an open access article distributed under the terms and conditions of the Creative Commons Attribution (CC BY) license (http://creativecommons.org/licenses/by/4.0/).

Figure S1. Clustering of proteins that interact with HMGB1 in SKOV-3 and PC-3 cells. (**A**) in the nuclear fraction of SKOV-3, (**B**) in the nuclear fraction of PC-3, (**C**) in the cytoplasmic fraction of SKOV-3, (**D**) in the cytoplasmic fraction of PC-3. Color code in A and B is as follows: red, mRNA splicing, via spliceosome; dark blue, rRNA processing; green, ribonucleoprotein complex biogenesis; yellow, nuclear transport; pink, chromatin organization; purple cell division; brown, DNA repair; dark green, NuRD complex. Color code in C and D is as follows: red, SRP-dependent co-translational protein targeting to membrane; blue, cytoplasmic translation; green, regulation of translation; yellow, cell division; dark green, septins; pink, intermediate filament cytoskeleton organization.The interactome of HMGB1 partners, reflecting only experimental or data-base recorded interactions, has been constructed with STRING (https://string-db.org/, Accessed 10 February 2021).

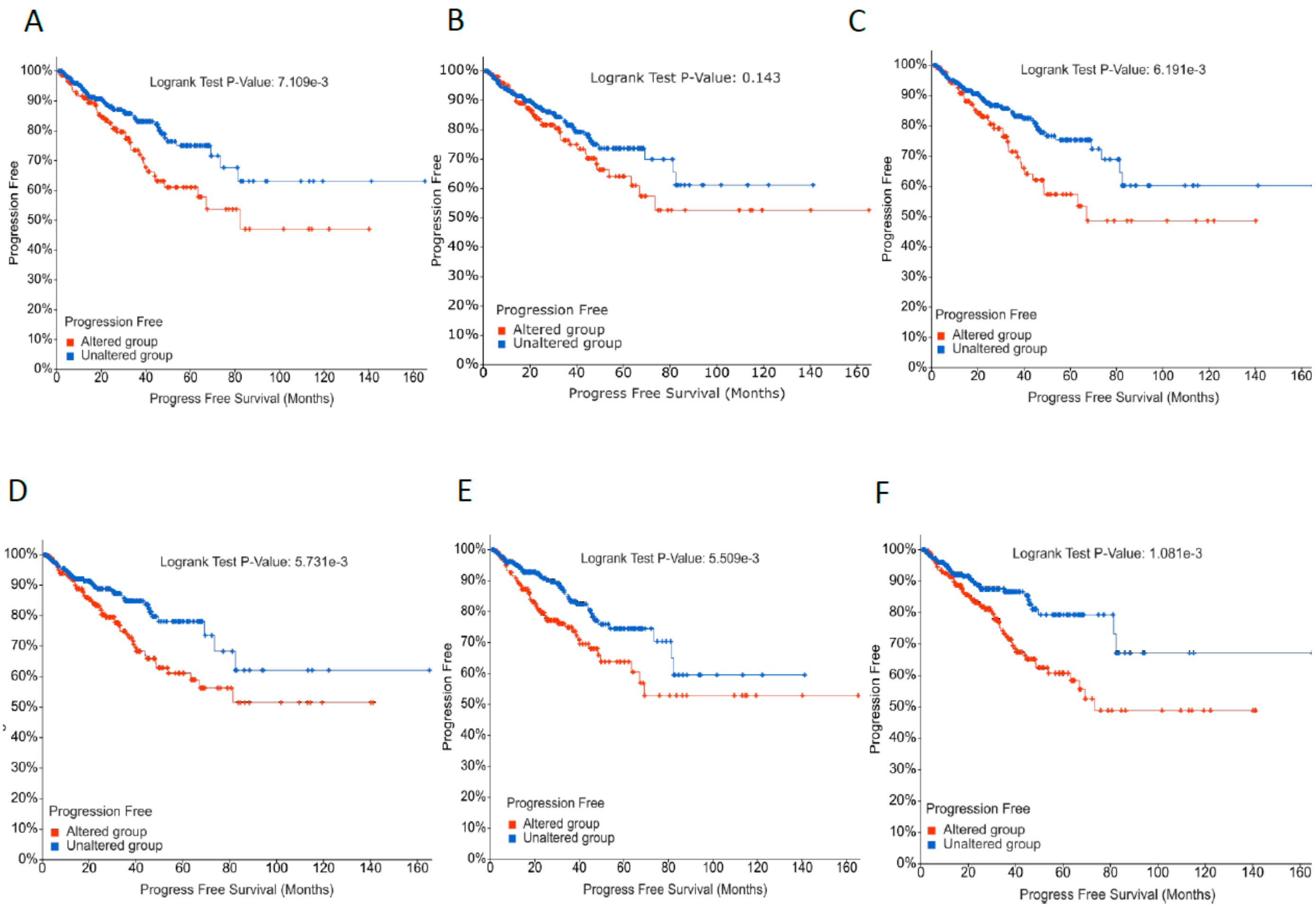

Figure S2. Correlation between high expression of functional groups of genes related to the PC-3 HMGB1 interactome and progression free survival in prostate adenocarcinoma patients. (**A**) LSU rRNA processing; (**B**) SSU rRNA processing; (**C**) 5.8 rRNA processing; (**D**) Ribonucleoprotein complex assembly; (**E**) spliceosome catalytic step 2; (**F**) nucleo-cytoplasmic transport.

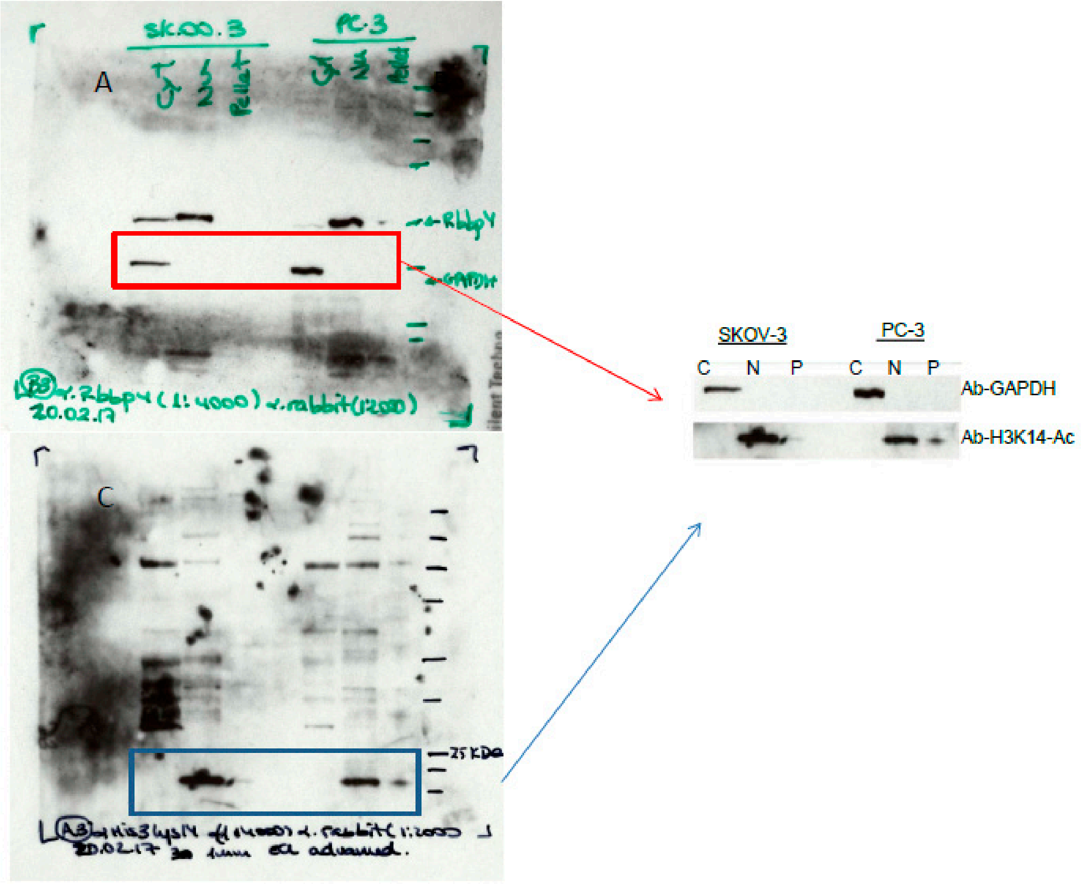

Original Blots Fig. 1A

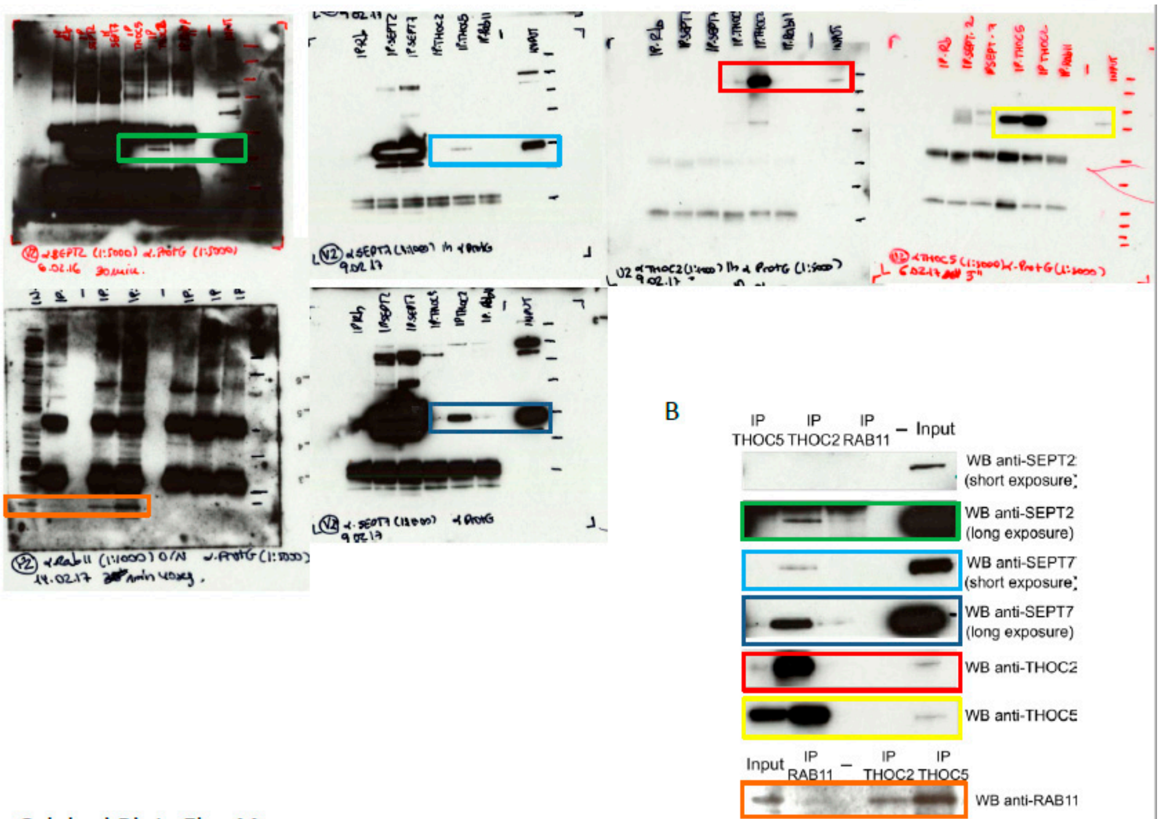

Original Blots Fig. 4A

Figure S3. Original Western Blot.
